# Assessing the pedestrian response to urban outdoor lighting: A full-scale laboratory study

**DOI:** 10.1371/journal.pone.0204638

**Published:** 2018-10-04

**Authors:** Johan Rahm, Maria Johansson

**Affiliations:** Environmental Psychology, Department of Architecture and Built Environment, Lund University, Lund, Sweden; Beihang University, CHINA

## Abstract

This study identifies and applies methods for evaluating the human response to pedestrian lighting applications intended for future use by the municipality of Malmö, Sweden. The methods employed provide a supplementary perspective to that given by the photometric properties of the lighting applications. The study involved 89 participants from two age groups (Young: N: 43, 19–31 yrs.; Elderly: N: 46, 62–77 yrs.). Data were collected in a full-scale laboratory using a mock-up pedestrian pathway. Three lighting applications (one ceramic metal halide and two LED) were presented and the participants’ behavior (walking speed), perception (ability to perform visual tasks–recognize facial expressions, detect obstacles, read street signpost), affective response, and evaluation of the lighting quality were assessed.

The three lighting applications significantly differed with regard to the human response. The facial expression recognition distance, sign reading distance and the obstacle detection task, along with the evaluation of lighting quality and level of arousal, distinguished one of the LEDs (Correlated Color Temperature: 3810, Color Rendering Index: 75, Scotopic/Photopic ratio: 1.48) from the other two lighting applications–the participants performed better on the visual tasks, and the lighting was perceived as brighter, more arousing and less pleasant. Methods to capture human perception, evaluation and behavior in relation to outdoor lighting, provide a valuable perspective that should be systematically applied when municipalities consider different pedestrian lighting applications.

## 1. Introduction

In the Nordic countries, daylight hours are very limited in winter. This makes pedestrians dependent on outdoor lighting for ensuring functional levels of visual accessibility and perceived safety when getting to and from work and while going about their everyday activities. Several studies support the importance of outdoor lighting for the walkability of a neighborhood [1–4] and it has been found to increase the level of walking after dark among all age groups [5–11].

However, the advantages of artificial lighting come at a cost, both environmental and financial. There is potential for reducing this cost through saving between 30–50% of the total annual lighting energy use [12] by updating existing outdoor lighting installations in terms of design and more energy-efficient light sources [13, 14]. When updating existing lighting applications, both technical and human aspects should be considered, to find pedestrian-friendly lighting solutions that are adapted to user needs while minimizing energy use. Special consideration must be taken to users from vulnerable groups, such as the elderly and the visually impaired [15].

In Sweden, many municipalities are on the verge of updating their outdoor lighting infrastructure, due to the age and state of existing installations, as well as for energy-conservation reasons [16]. In view of the sheer scale of the investments, much is won if the human perspective is applied early in the decision-making process.

The technical aspects of lighting have a set of standard measures stipulated in national and international standards [17–19]. However, there is to date no consensus on measures to capture pedestrians’ response to outdoor lighting. This study, part of the EU project Lighting Metropolis [20] and a collaboration with the City of Malmö, evaluates the human response to three different outdoor lighting applications proposed by the city authority for future use in pedestrian environments in Malmö. In order to employ a within-subjects design with a wide variety of measures and in order to reduce confounding factors (such as traffic, other pedestrians and weather), a mock-up in a laboratory was used.

### 1.1 Previous research

Using a systematic literature review of the research on the human response to outdoor lighting [21] as a starting point, the study set out to assess differences between lighting applications in terms of the human response.

**Fig 1:**
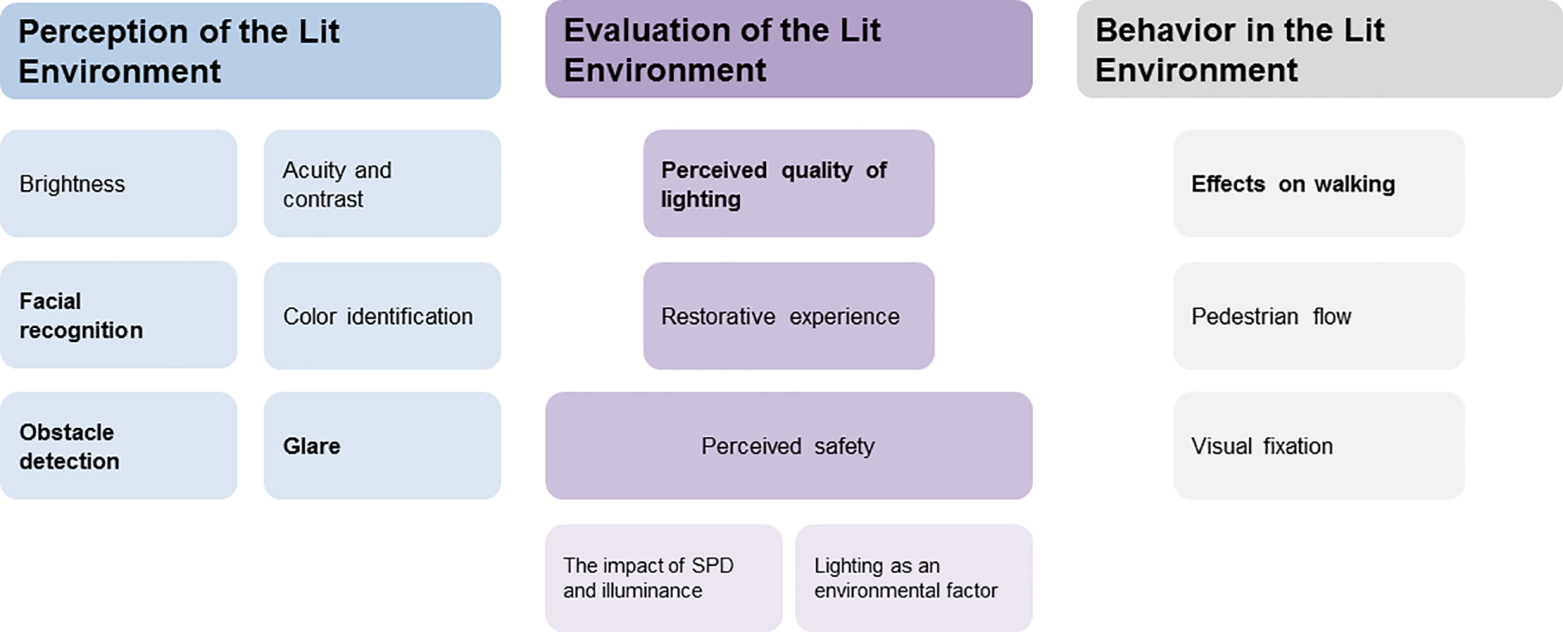
Overarching themes and corresponding categories identified through the systematic literature review. Categories included in the study are marked in bold.

The systematic review suggested that three major themes of human responses were relevant for the design of new energy-efficient pedestrian-friendly lighting installations. The first theme, ‘Perception of the lit environment’, concerns how light is perceived differently depending on individual factors, such as age and eyesight, and on the various characteristics of the light source, especially at mesopic vision. The second and third themes concern how the individual responds to the perceived environment, either psychologically, ‘Evaluation of the lit environment’, or physically, ‘Behavior in the lit environment’ [21] (Fig 1). Methods associated with each theme proposed in the literature and considered potentially feasible for evaluating outdoor lighting are described below.

Within the theme human perception of the lit environment the proposed tasks related to facial recognition and obstacle detection were deemed feasible for use in a practical setting, so were included in the laboratory study. Facial recognition is considered to be an important visual task for pedestrians after dark, since judging the intent of oncoming pedestrians from a safe distance is deemed important for the perception of safety [22]. This has been investigated both in laboratory settings [23–28] and in the field [14, 15, 29–33], using photographs, dummies or assistants as targets that should be recognized by the respondents. A common technique has been to measure the distance at which the respondents are certain of the gender, can guess the identity, or make out facial features. It is suggested, however, that recognition of facial *expressions* may be a more relevant task for evaluating lighting quality, and more ecologically valid due to its greater relevance for judging the intent of oncoming strangers [34].

The second task, obstacle detection, has been explored in the laboratory using apparatus with adjustable obstacle heights [35, 36] and by using a mock-up path with a false floor displaying obstacles at various heights [37, 38]. An inverse relationship has been found between illuminance level and the obstacle height needed for it to be detected, and at low light levels (0.2 lx) younger participants detect significantly lower obstacles than older participants [35, 37]. Also, at equally low light levels, lighting applications with higher scotopic/photopic (S/P) ratios consistently assisted detection of lower obstacles. At higher illuminance levels (2 & 20 lx) the differences due to age and Spectral Power Distribution (SPD) were non-significant [35, 37].

Within the second overarching theme, the evaluation of the lit environment, perceived lighting quality and the impact of lighting on the emotional state were selected for use in the laboratory. Perceived lighting quality adds the human evaluation component to the assessment of lighting quality and complements the technical environmental assessment measures. It has been conceptualized in different ways [14, 15, 39–43]. In a recent effort to assess the perceived outdoor lighting quality, Johansson et al. [15] and Kuhn et al. [14] used the dimensions brightness and hedonic tone, previously used by Küller and Wetterberg [44, 45] to describe subjective impressions. Their concept has since been further developed by the development of the Perceived Outdoor Lighting Quality scale (POLQ) [39], which distinguishes between lighting applications on two indices, the Perceived Strength Quality (PSQ) and the Perceived Comfort Quality (PCQ) index. In addition to the more readily accessible qualitative evaluation of lighting properties, it has also been found that lighting may affect the emotional state of the individual, regarding the level of arousal and pleasure [43, 46, 47].

Within the third overarching theme, behavior in the lit environment, walking speed was deemed to be a measure with potential to differentiate between the three lighting applications. Studies indicate that illuminance level may have tangible effects on pedestrian behavior. Insufficient lighting is suggested to decrease walking speed [48, 49] and make people look at the ground more, compared to brighter conditions [50].

### 1.2 Aim and hypotheses

The aim of this study was to assess differences between three lighting applications fulfilling requirements for current Swedish standards on illuminance levels at pedestrian paths but differing in photometric qualities with regard to light distribution, Correlated Color Temperature (CCT), SPD and glare. The lighting applications were chosen on the basis of being considered for future use at pedestrian paths in the city of Malmö, Sweden.

The three overarching themes of human perception, evaluation and behavior in response to artificial outdoor lighting were addressed, focusing on the previously researched aspects that were feasible to use in an outdoor setting and that would be relevant and of practical use in the decision-making processes of municipalities. Perception involved obstacle detection, facial recognition, sign reading; Evaluation involved perceived quality of lighting, emotional state; and for Behavior, walking speed was measured.

To identify lighting applications suitable for all age groups, a further aim was to test whether people from different age groups responded differently to the three lighting applications.

## 2. Method

### 2.1 Participants

The study comprised 89 participants divided into two age groups: one group of young people (N: 43, aged 19–31 years, mean age: 22, 47% female) and one group of elderly (N: 46, aged 62–77, mean age: 69, 54% female). The participants were recruited through information meetings at organizations for the elderly, in public places, on the university campus and through personal networks. The participants’ visual acuity and contrast vision were tested using Sloan optotypes on a visual acuity board (by Precision Vision) at a distance of 3 m. When applicable, participants wore the same glasses as normally worn outdoors. Color vision was tested with the Ishihara Color Vision Test (Young: Acuity both eyes: min = 0.77 max = 1.25, mean = 1.21; Contrast: min = 0.0 max = 0.5, mean = 0.46; Full color vision 98%; Elderly: Acuity both eyes min = 0.46, max = 1.25, mean = 1.00; Contrast min = 0.0 max = 0.5, mean = 0.30, Full color vision 85%).

### 2.2 Ethics statement

This study was carried out in accordance with the rules and regulations laid down by the Ethics Committee for the Swedish Research Council [51] after consultation with the Regional Ethical Review Board. The Board concluded that approval according to the Swedish Ethical Review Act was not needed for this study. Information about the aim of the study was given and written informed consent was obtained from all participants in accordance with the Declaration of Helsinki. The participants were informed of their right to withdraw at any time without giving an explanation. Personal information was anonymized to retain the privacy of the participants, who received approximately 52 EUR after participation as remuneration.

### 2.3 Setting and lighting applications

**Fig 2:**
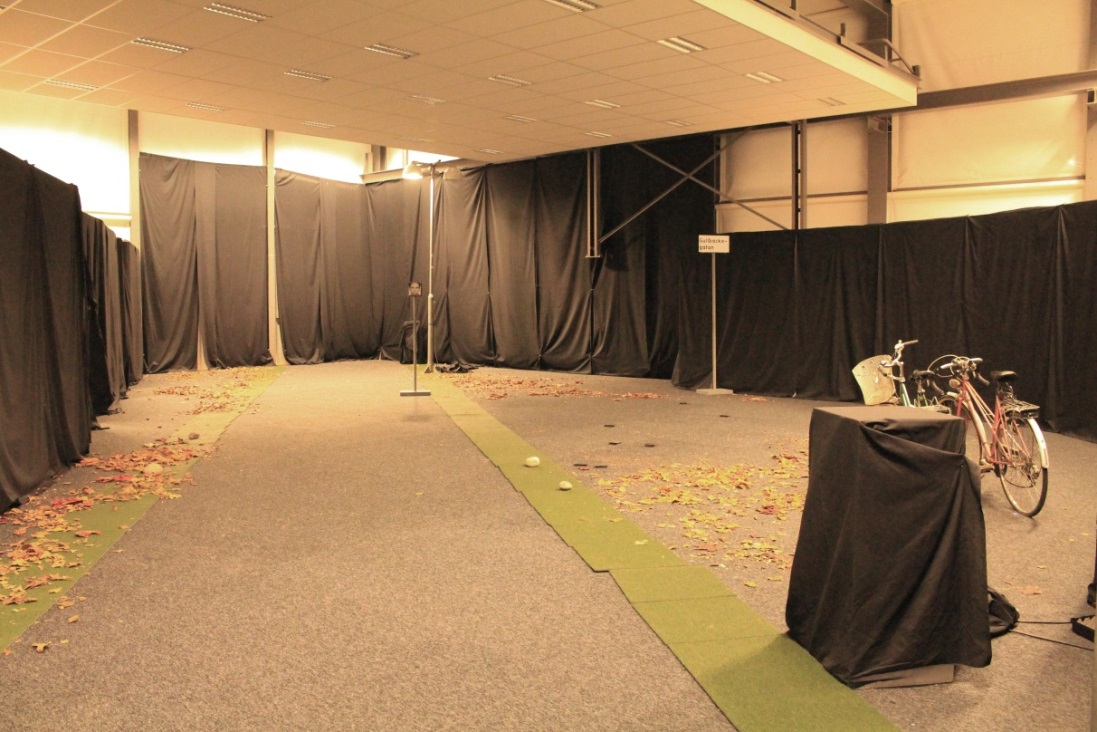
Photograph of the mock-up pathway (placed diagonally in the full-scale laboratory) under lit conditions.

The study was carried out in a laboratory (14.5 x 18.5 x 4.5 m) where a 23-meter long and 2.5-meter wide pedestrian path was constructed along the diagonal axis, with two lampposts with the height of 4 meters placed 16 meters apart (1.5 and 17.5 meters from the starting point) (Fig 2). The lampposts were of the same height as the standard for Malmö municipality, but were placed closer together than the normal 21 meters. The walls were covered in black cloth and the floor had a graphite-grey carpet with a reflection factor of 5%. Adjacent to the laboratory was a waiting room with a lighting design resembling that of an apartment in Sweden (${\bar{E}}_{H}:292\;lx$). The participants were thus not dark-adapted when they entered the laboratory. The scenario was designed to imitate the process of going outside from a brightly lit home in the evening.

**Fig 3:**
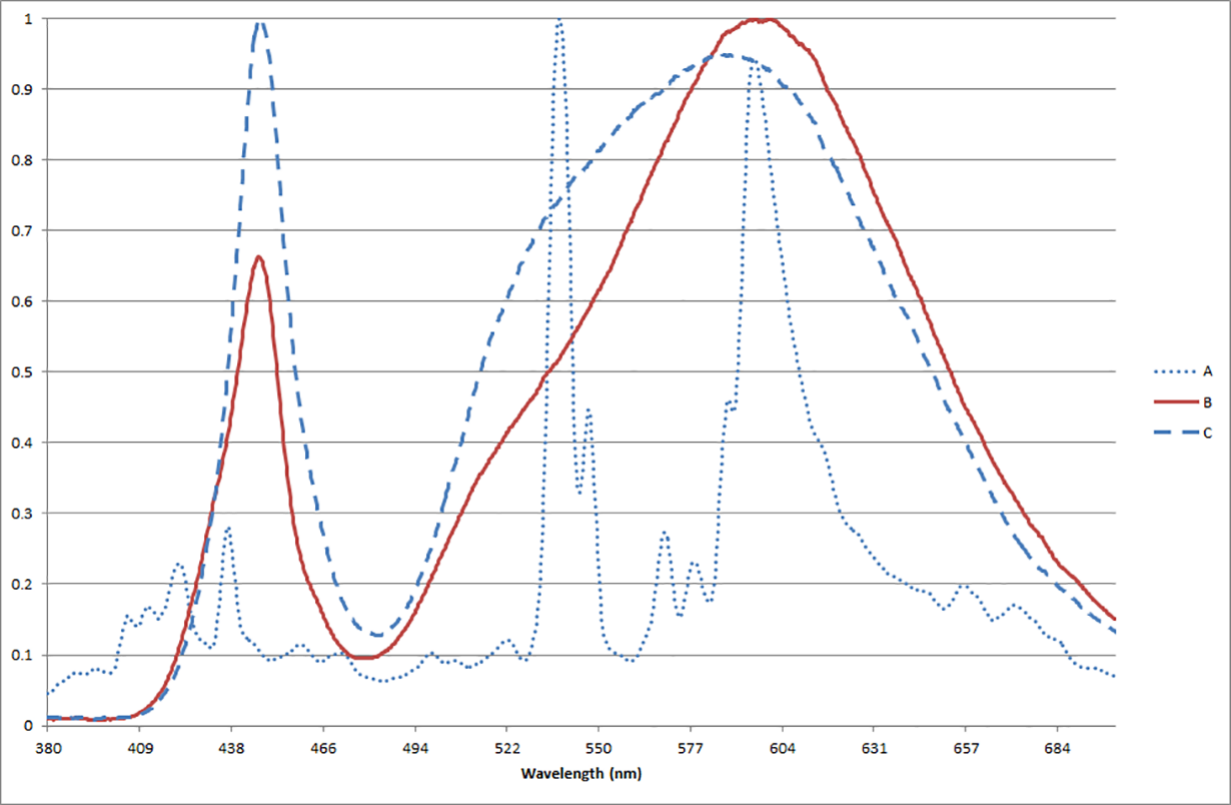
Normalized SPD for the three lighting applications. Relative power is depicted on the y-axis and wavelength on the x-axis.

**Fig 4:**
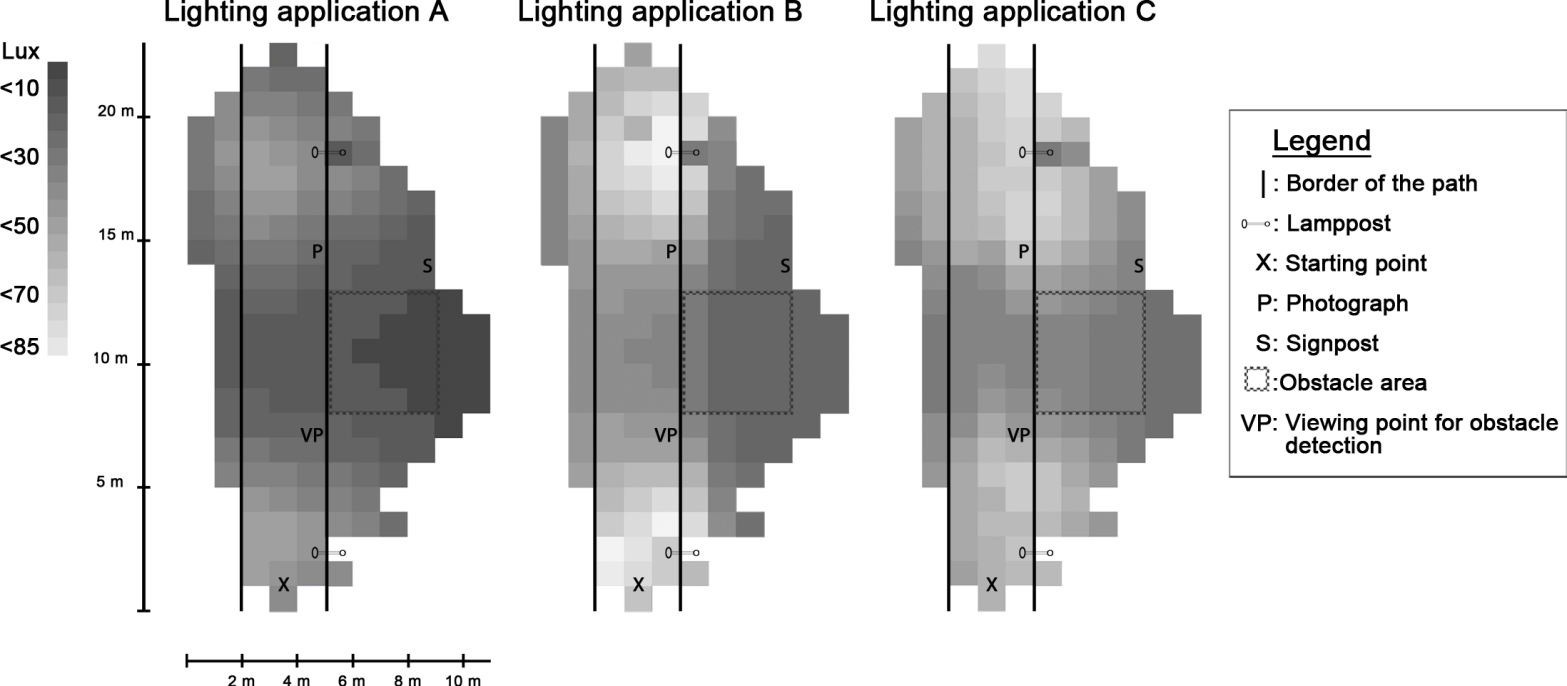
Gradient maps illustrating the horizontal illuminance for lighting applications A, B and C, border of the path, location of lampposts, starting point, obstacle detection area and locations of the photograph and street signpost. Illuminance was measured using a grid of 1x1 m. The grid followed the contours of the irregularly shaped laboratory.

**Fig 5:**
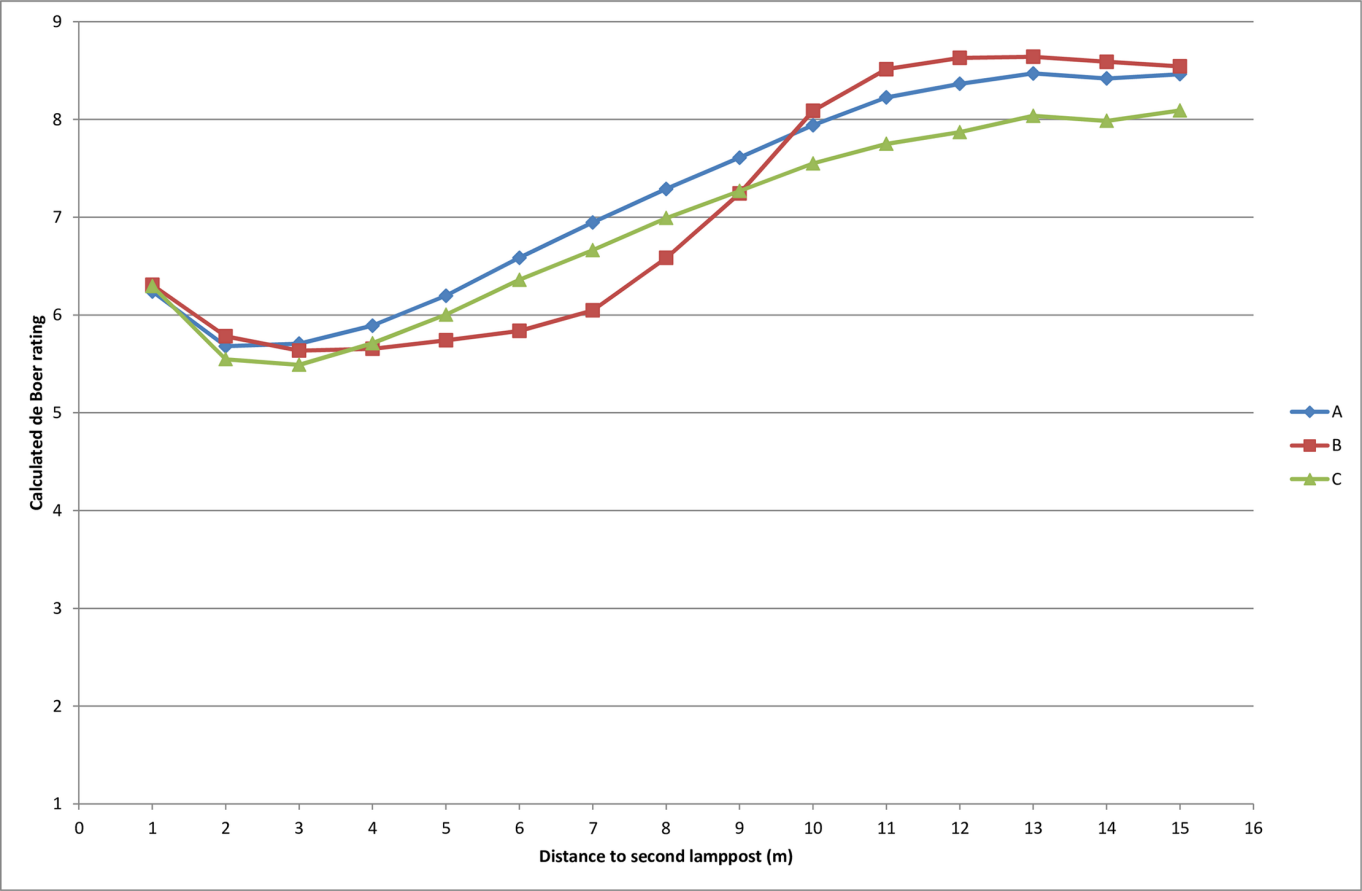
Discomfort glare de Boer rating calculations based on vertical illuminance by the light source, vertical illuminance by ambient light and the horizontal viewing angle. (de Boer scale: 1 = unbearable, 3 = disturbing, 5 = just permissible, 7 = satisfactory, 9 = just noticeable).

The three lamps were one ceramic metal halide (Lighting application A) and two LEDs (Lighting applications B & C) (Table 1, for SPD see Fig 3). The differences in light distribution are manifested in the horizontal illuminance gradient maps (Fig 4). The horizontal illuminance (E_H_) on the path varied between 7 and 86 lx (Lighting application A, E_H_: 7–45 lx, ${\bar{E}}_{H}$: 24 lx; Lighting application B, E_H_: 17–86 lx, ${\bar{E}}_{H}$: 46 lx; Lighting application C, E_H_: 14–67 lx, ${\bar{E}}_{H}$: 41 lx). The discomfort glare de Boer rating was calculated for each lighting application based upon the vertical illuminance at the eye provided by the glare source (E_g_), the vertical illuminance at the eye provided by ambient light (E_b_),and the horizontal viewing angle between the viewing direction and the luminaire (Ɵ), following the model proposed by Lin et. al. [52] (Eq 1, Fig 5). E_g_ was measured every meter, in the middle of the path, at a height of 1.5 meters. E_b_ was estimated by calculating the average of three measures of the vertical illuminance reflected from the walls of the laboratory.

$$de\;Boer\;rating=7.09-{log}_{10}(\frac{E_{g}^{2.21}}{E_{b}^{1.02}\;x\;{Ɵ}^{1.62}})$$ (1)

**Table 1:** Technical specifications for the lighting applications.

Lighting application	A	B	C
Light source	CMH	LED	LED
Power (W)	68	36	93
Luminous efficacy (lm/W)	50	94	64
Path mean horizontal illuminance (lx)	24	46	41
Laboratory mean horizontal illuminance (lx)	18	28	32
S/P	1.25	1.22	1.48
CCT (K)	2890	2912	3810
Color rendering index, CRI	81	75	75
Face luminance (cd/m^2^)	0.28	0.36	0.55
Sign luminance (cd/m^2^)	0.13	0.13	0.35
Obstacle illuminance range (lx)	4–8	2–15	10–21

### 2.4 Measurements

#### 2.4.1 Perception

Three tasks were used to evaluate visual accessibility: obstacle detection, facial expression recognition, and sign reading.

The obstacle detection task was inspired by the laboratory studies by Fotios and Cheal [35, 36]. The participants were placed at a specific viewing point and instructed to focus straight ahead while simultaneously stating the number of obstacles (10 x 10 x 2.5 cm, made of the same carpet as was covering the floor) they could discern on the ground on their right-hand side. The size and height of the obstacles were chosen to try to emulate a raised cobblestone [36]. The obstacle detection area was shielded by the researcher’s body while the participants walked towards the viewing point. To help prevent participants from glancing at the obstacles, the researcher maintained eye contact with the participants while giving instructions for the task.

**Fig 6:**
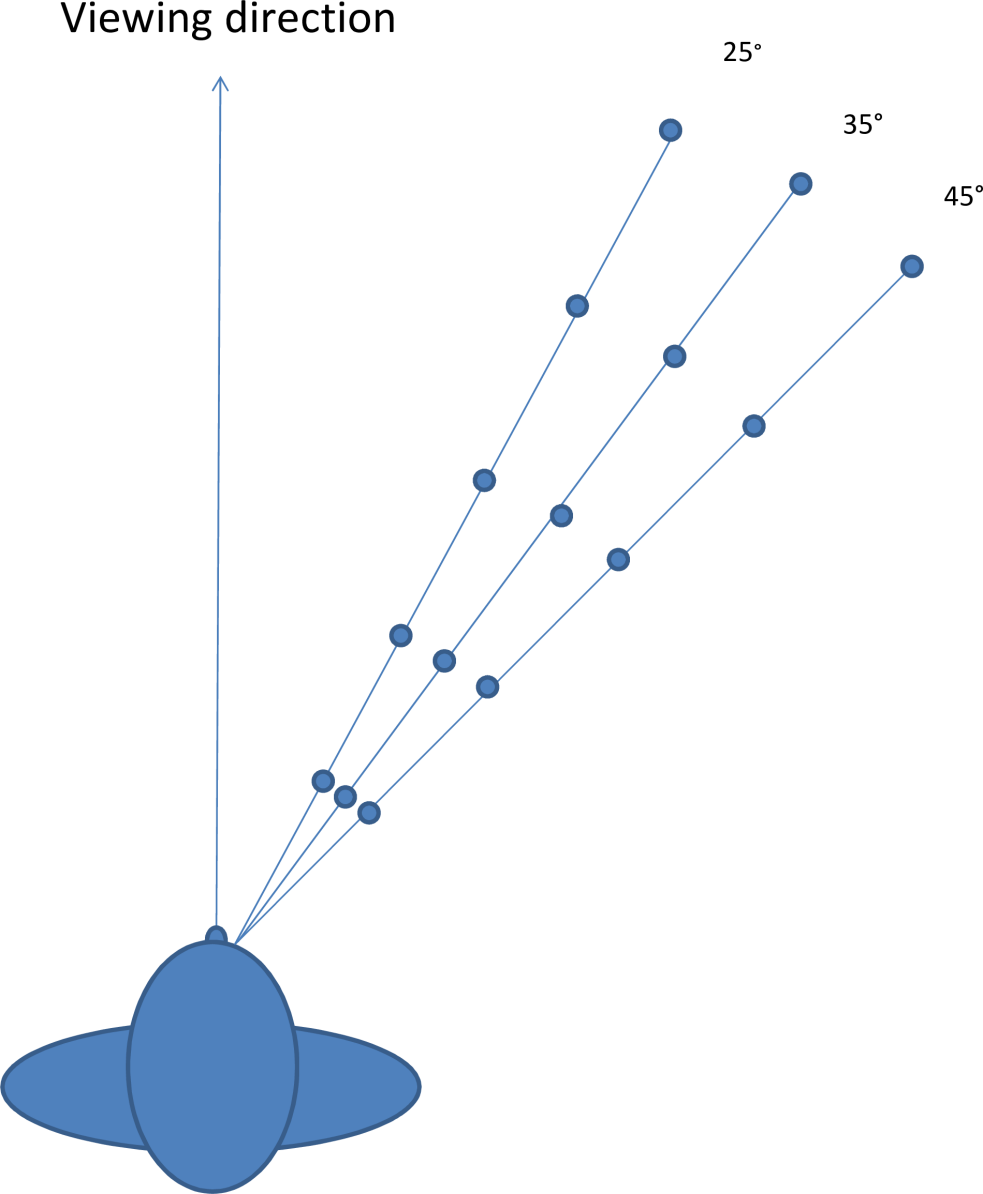
The 15 possible positions for the obstacles in the obstacle detection task.

For each presentation the number of obstacles was chosen randomly and varied between 4 and 7. The position of the obstacles was also chosen randomly from a pool of 15 positions created by using a 25, 35 and 45 degree angle from the viewing direction and at distances representing 2, 4, 6, 8 and 10 paces from the participants (corresponding to 1.2, 2.4, 3.6, 4.8 & 6 meters [36], see Fig 6). The size of the obstacles, given as visual angle subtended at the eye, ranged from 4.77° at 1.2 meters to 0.95° at 6 meters distance.

The illumination measurements of the obstacle area (4 x 5 meters, located on the right-hand side of the path, centered on the midpoint of the distance between the lampposts, see Fig 4.) were conducted as part of the overall illumination measurement, using a 1x1-meter grid. The horizontal illuminance measurements for the obstacle detection area varied between 2 and 33 lx (Lighting application A, E_H_: 3–10 lx, ${\bar{E}}_{H}$: 6 lx; Lighting application B, E_H_: 2–17 lx, ${\bar{E}}_{H}$: 5 lx; Lighting application C, E_H_: 11–33 lx, ${\bar{E}}_{H}$: 17 lx) with the corresponding uniformities U_A_: .59, U_B_: .28 and U_C_: .63. Horizontal illuminance was also measured at the center of each obstacle, with measurements ranging from 2 to 15 lx (Lighting application A, E_H_: 4–8 lx, ${\bar{E}}_{H}$: 5 lx; Lighting application B, E_H_: 2–15 lx, ${\bar{E}}_{H}$: 6 lx; Lighting application C, E_H_: 10–21 lx, ${\bar{E}}_{H}$: 15 lx).

In the facial expression recognition task, the participants were instructed to walk towards a photograph of a woman´s face (175x200 mm; positioned at a height of 1.65 m; printed on non-glossy paper), placed on the right-hand side of the path 12 meters from the first lamppost (13.5 meters from the starting point). The participants were instructed to stop when they could discern the facial expression of the woman’s face. The facial photographs belong to the standardized P. Ekman’s Emotions Revealed photo set, and the photos used were fear (p. 165), surprise (p. 166) and anger (p. 127) [53].The distance was measured, and the correctness of the evaluation was assessed on 11 items (Interest, Enjoyment, Surprise, Anger, Disgust, Contempt, Fear, Shyness, Shame, Guilt & Hostility) from the Differential Emotions Scale, DES [54], graded using a 5-point Likert scale. Since the presentation order of the lighting applications was counterbalanced, not all participants experienced the same facial expression/lighting application combination. To overcome this difference the mean value for the three DES items that corresponded with the facial expressions of the photographs (fear, surprise and anger) was calculated and subtracted with the overall mean rating for all DES items. This was done for each lighting application, and the resulting variable was then used as a proxy for the ability to identify the relevant facial expressions.

In the sign reading task, the participants were asked to walk along the path towards a street signpost placed 4 meters to the right of the path and 4.5 meters before the second lamppost (13.6 meters from the starting point), positioned at a height of 2.10 meters. Three versions of the street signpost were used, each showing street names of similar type and equivalent number of syllables (Gullbackegatan, Almedalsgatan & Hagalundsgatan; Tratex font; Size: 212). The task of the participants was to stop when the text on the street signpost became legible. The distance was measured and the participants were asked to read the street name aloud in order to validate the legibility.

#### 2.4.2 Evaluation

In order to assess the emotional state of the participants when they had just finished walking down the path, the affect grid [55, 56], an instrument in which the participants rate their degree of arousal and valence in a two-dimensional grid (consisting of 5 x 5 cells with the labels Active placed above, Passive below, Negative to the left and Positive to the right), was used. Two other scales, asking the participants to rate their valence (1 = sad, depressed, displeased to 5 = glad, happy, pleased) and arousal-level (1 = dull, passive, sleepy to 5 = peppy, active, awake), were administered. The mean score for the arousal measures (α_A_ = .874; α_B_ = .900; α_C_ = .859) and valence measures (α_A_ = .904; α_B_ = .846; α_C_ = .690) was then calculated.

To capture how the participants experienced the lighting and the lit environment, the POLQ scale [39] was used, which consists of ten items (subdued-brilliant; strong-weak; dark-light; unfocused-focused; clear-drab; hard-soft; warm-cool; natural-unnatural, glaring- shaded; mild-sharp) rated on a 7-point scale, constituting two indices, PSQ and PCQ. The participants were also asked to rate how well they could see under the present lighting application using a 7-point Likert scale (1 = very poorly, to 7 = very well).

#### 2.4.3 Behavior

Behavior was, in line with Pedersen and Johansson [48], assessed in terms of walking speed. Before entering the laboratory, the baseline walking speed of the participants was measured on a 33.3-meter distance in a corridor under well-lit conditions (${\bar{E}}_{H}=195\;\mathrm{lx}$). Then, in the laboratory, at the start and end of the path, motion sensors connected to a stopwatch measured the time it took the participants to walk the path, and walking speed was calculated. The impact of lighting on walking speed was then calculated for each lighting application by subtracting the walking speeds from the laboratory from the baseline walking speed.

### 2.5 Design and procedure

The study employed a mixed design, with a within-subjects repeated measures design for evaluating differences due to the different lighting applications, and a between-groups design for exploring differences due to age. Each participant performed the test procedure three times, once for each lighting application (Table 2). The luminaires were shifted by rotating the top of the lamppost and the presentation order was counterbalanced. The participants arrived in groups of five and, before entering the lab, they gathered in a waiting room, where they were informed about the procedure and asked to fill in questionnaires surveying background data and individual characteristics. In an adjacent room their vision was tested individually and then they walked a 33-meter distance in a corridor where their baseline walking speed was measured.

**Table 2:** Flowchart for the test procedure.

	Process	Location
	Introductory meeting	Waiting room
	Measurement of baseline walking speed	Corridor
	Vision tests	
Lighting presentation #1		Laboratory
	Measurement of walking speed	
	Affect grid	
	Valence & arousal scales	
	POLQ	

	Waiting / self-report of background data	Waiting room
		Laboratory
	Obstacle detection	
	Facial recognition	
	Sign reading	

	Waiting	Waiting room
Lighting presentation #2		Laboratory
	Measurement of walking speed	
	Affect grid	
	Valence & arousal scales	
	POLQ	

	Waiting	Waiting room
		Laboratory
	Obstacle detection	
	Facial recognition	
	Sign reading	

	Waiting	Waiting room
Lighting presentation #3		Laboratory
	Measurement of walking speed	
	Affect grid	
	Valence & arousal scales	
	POLQ	

	Waiting	Waiting room
		Laboratory
	Obstacle detection	
	Facial recognition	
	Sign reading	

	Debriefing and conclusion	Waiting room

The participants entered the laboratory one at a time, while the rest of the group remained in the waiting room. The participants were asked to walk down the path past the second lamppost, after which a podium was placed where the participants rated their emotional state. The participants then returned to the first lamppost, looked out over the path and rated the perceived lighting quality [39].

When all participants had completed the task, they waited in the adjoining waiting room while the visual performance tasks were prepared. Next, the participants individually entered the laboratory a second time and walked to the obstacle detection point 5.5 meters down the path from the first lamppost and performed the obstacle detection task. The participants returned to the starting point before starting the facial expression recognition task, after which they returned to the starting point. Lastly, the participants performed the sign reading task. Data collection took approximately two hours for each group of participants.

The data was analyzed with IBM SPSS 22, using Repeated Measures ANOVA with age as between-subjects factor. To avoid potential problems with violations of assumptions underlying the use of ANOVA, parallel analyses were conducted using a two-way between-within subjects ANOVA on the trimmed means (20%) using the bwtrim function of R. The results from the trimmed means ANOVA corroborated the findings from the Repeated Measures ANOVA.

## 3. Results

### 3.1 Perception

**Fig 7:**
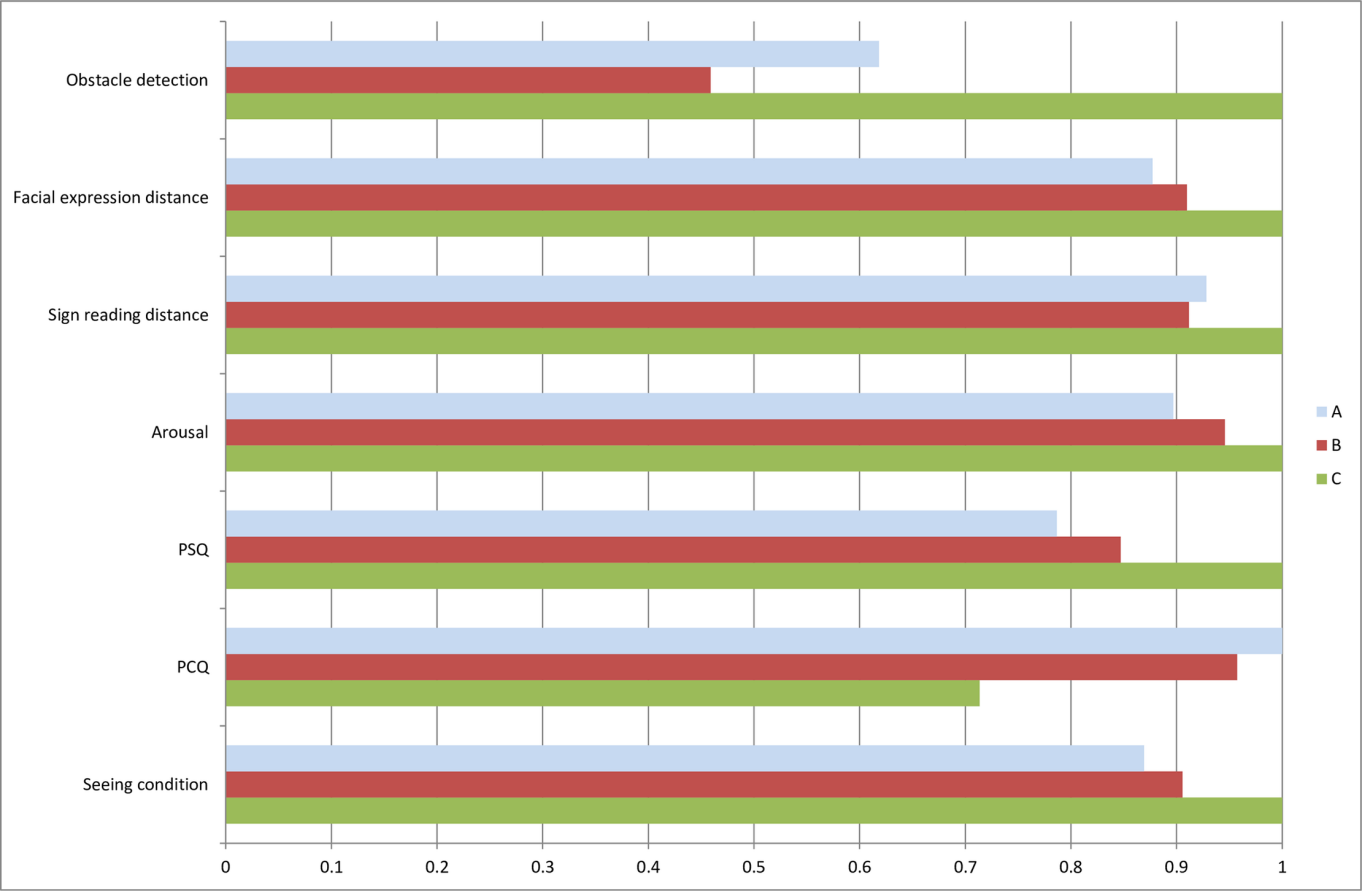
Overview of significant results in Table 3 (each measure rescaled by division by its greatest value).

There were statistically significant differences between the different lighting applications for obstacle detection, facial expression distance, and sign reading distance (Table 3, Fig 7). For obstacle detection, participants were most successful in detecting obstacles under lighting application C, on average detecting 42% of the obstacles, followed by lighting application A (26%), and then lighting application B (19%) (F (2, 174) = 38.021, p = .000, η_p_^2^ = .304). Similar results were found for the distances needed to recognize a facial expression (F (2, 174) = 8.115, p = .000, η_p_^2^ = .085) and the distance to read the street signpost (F (2, 174) = 31.906, p = .000, η_p_^2^ = .268). Lighting application C enabled the participants to identify facial expressions and read a street signpost at significantly greater distances (mean distance, $\bar{d}$: 4.6 & 11.3 meters) than lighting applications A ($\bar{d}:$ 4.0 & 10.1 meters) and B ($\bar{d}:$ 4.2 & 9.9 meters) (Table 4).

**Table 3:** Results from the repeated measures ANOVA.

Response	Within subject	Between groups	Interaction	Post hoc*
Perception				
*Obstacle detection*	F(2, 174) = 38.021,	F(1, 87) = 10.234,	F(2, 174) = 1.164,	C > A > B
	**p = .000**, η_p_^2^ = .304	**p = .002**, η_p_^2^ = .105	p = .315	
*Facial expression distance*	F(2, 174) = 8.115,	F(1, 87) = 7.291,	F(2, 174) = 3.257,	C > A & B
	**p = .000**, η_p_^2^ = .085	**p = .008**, η_p_^2^ = .077	**p = .041**, η_p_^2^ = .036	
*Facial expression recognition*	F(2,174) = .791,	F(1, 87) = 7.437,	F(2, 174) = .788,	—
	p = .455	**p = .008**, η_p_^2^ = .079	p = .456	
*Sign reading distance*	F(2, 174) = 31.906,	F(1, 87) = 74.308,	F(2, 174) = 1.292,	C > A & B
	**p = .000**, η_p_^2^ = .268	**p = .000**, η_p_^2^ = .461	p = .277	
Evaluation				
*Arousal*	F(2, 174) = 10.084,	F(1, 87) = 12.793,	F(2, 174) = 2.437,	C > A
	**p = .000**, η_p_^2^ = .104	**p = .001**, η_p_^2^ = .128	p = .090	
*Valence*	F(2, 174) = 1.903,	F(1, 87) = 27.695,	F(2, 174) = .927,	—
	p = .152	**p = .000**, η_p_^2^ = .241	p = .398	
*PSQ*	F(2, 174) = 44.148,	F(1, 87) = .089,	F(2, 174) = 4.827,	C > A & B
	**p = .000**, η_p_^2^ = .337	p = .766	**p = .009**, η_p_^2^ = .053	
*PCQ*	F(2, 174) = 33.617,	F(1, 87) = 20.693,	F(2, 174) = 4.074,	A & B > C
	**p = .000**, η_p_^2^ = .279	**p = .000**, η_p_^2^ = .192	**p = .019**, η_p_^2^ = .045	
*Seeing condition*	F(2, 174) = 12.892,	F(1, 87) = .330,	F(2, 174) = 1.656,	C > A & B
	**p = .000**, η_p_^2^ = .129	p = .567	p = .194	
Behavior				
*Walking speed difference*	F(2, 174) = 1.427,	F(1, 87) = 1.606,	F(2, 174) = .131,	—
	p = .243	p = .208	p = .877	

* Bonferroni, p < .017

**Table 4:** Mean scores and standard deviations for all measures, by lighting application and age group.

			Mean (SD)			
	Lighting A		Lighting B		Lighting C	
Response	Young	Old	Young	Old	Young	Old
Perception						
*Obstacle detection*	.33 (.23)	.20 (.23)	.24 (.22)	.16 (.21)	.51 (.21)	.35 (.30)
*Facial expression distance*	4.28 (1.40)	3.73 (2.10)	4.81 (1.75)	3.53 (2.04)	5.08 (1.72)	4.07 (1.87)
*Facial expression recognition*	.37 (.50)	.06 (.48)	.38 (.62)	.20 (.33)	.33 (.63)	.16 (.39)
*Sign reading distance*	11.83 (1.90)	8.47 (2.24)	11.66 (1.91)	8.29 (2.16)	12.80 (1.40)	9.08 (2.37)
Evaluation						
*Arousal*	3.40 (.94)	4.08 (.91)	3.73 (.92)	4.16 (.72)	4.05 (.73)	4.31 (.57)
*Valence*	3.52 (.84)	4.10 (.83)	3.70 (.98)	4.28 (.59)	3.53 (.80)	4.34 (.59)
*PSQ*	4.28 (1.08)	4.50 (1.27)	4.66 (1.01)	4.80 (1.19)	5.86 (.70)	5.33 (.97)
*PCQ*	4.09 (1.25)	4.77 (1.09)	4.13 (1.35)	4.37 (1.25)	2.55 (.81)	3.75 (1.23)
*Seeing condition*	5.07 (1.44)	5.51 (1.38)	5.58 (1.12)	5.46 (1.24)	6.10 (.95)	6.09 (1.09)
Behavior						
*Walking speed difference*	.17 (.13)	.20 (.15)	.15 (12)	.19 (.15)	.15 (.12)	.19 (.18)

For obstacle detection, there were significant differences between the two age groups, where the younger group performed significantly better on average (36%) than the older group (24%) (F (1, 87) = 10.234, p = .002, η_p_^2^ = .105). Also, the younger group could both identify facial expressions ($\bar{d}:$ 4.7 meters) and read the street signpost ($\bar{d}:$ 12.1 meters) at greater distances than the older group (Table 3). For facial expression distance there was also a weak interaction effect, indicating that the older group needed to be closest for lighting application B (F(2, 174) = 3.257, p = .041, η_p_^2^ = .036), whereas the younger group had their shortest distance for lighting application A. The younger participants were also more accurate in their assessment of facial expressions (F (1, 87) = 7.437, p = .008, η_p_^2^ = .079).

### 3.2 Evaluation

Instruments used for evaluating the lighting and the lit environment also showed significant differences. For the perceived lighting quality indices, PSQ and PCQ, there were significant differences between the three lighting applications (PSQ, F (2, 174) = 44.148, p = .000, η_p_^2^ = .337; PCQ, F (2, 174) = 33.617, p = .000, η_p_^2^ = .279). Lighting application C was rated highest on perceived strength quality (mean PSQ: 5.6) and lowest on perceived comfort quality (mean PCQ: 3.2). For the emotional state measurement, the different lighting applications differed on level of arousal (F (2, 174) = 10.084, p = .000, η_p_^2^ = .104), where lighting application C was experienced as more arousing than lighting application A, but not significantly different from B. When it came to judging how well they could see under the different lighting applications, lighting application C was deemed to give significantly better seeing conditions (F(2, 174) = 12.892, p = .000, η_p_^2^ = .129) (Table 3).

There were significant differences between the age groups for PCQ (F (1, 87) = 20.693, p = .000, η_p_^2^ = .192), where the younger group had lower average ratings (3.59 compared to 4.9), but not for PSQ F (1, 87) = .089, p = .766). However, for both indices there were interaction effects (PSQ, F (2, 174) = 4.827, p = .009, η_p_^2^ = .053; PCQ, F (2, 174) = 4.074, p = .019, η_p_^2^ = .045). For PSQ, lighting application C was rated higher by the younger group. For PCQ, lighting application B got higher ratings in the younger group, but still less than for the older group. Differences between the age groups were also found for emotional state. The older group was both more aroused (F (1, 87) = 12.793, p = .001, η_p_^2^ = .128) and felt more positive (F (1, 87) = 27.695, p = .000, η_p_^2^ = .241) than the younger group.

### 3.3 Behavior

Relative walking speed did not differ between the three lighting applications (F (2, 174) = 1.427, p = .243), and there were no significant differences between the different age groups (F (1, 87) = 1.606, p = .208).

## 4. Discussion

This study employed a set of methods for evaluating the human response to outdoor lighting and assessing differences between three lighting applications selected by the city of Malmö. The main finding is that the set of methods could differentiate between the three lighting applications with regard to the human response and thereby provide a complementary perspective to photometric properties. Most methods capturing perception and evaluation differentiated between lighting application C and the other two lighting applications (obstacle detection, facial recognition distance, sign reading distance, arousal, PSQ, PCQ and seeing condition). However, the behavior measure, walking speed, failed to capture any differences between the lighting applications.

The lighting applications differed in terms of the underlying technology and consequently also on several photometric measures, including light distribution, illuminance levels, SPD, CCT, CRI, and glare.

There were also differences in power and luminous efficacy between the lighting applications. Lighting application C used more than 2.5 times the power than lighting application B, which used the least power (P_A_:68 W P_B_: 36 W; P_C_:93 W). Lighting application B was also most efficient, having a luminous efficacy well above the other lighting applications (A: 50 lm/W; B: 94 lm/W; C: 64 lm/W).

This complexity regarding photometry, energy efficiency and, as result, the cost of operating the lighting applications is characteristic for the situation municipalities will face in any given procurement process. In addition to energy efficiency and road lighting standards, it is relevant to consider the human response in the choice of outdoor lighting applications for pedestrians. The methods used in this study could be applied during such a process as a way to differentiate between different lighting applications based on the human response to the lighting.

Most differences concerned lighting application C in relation to lighting applications A and B. The question remains why the measures did not substantially differentiate between lighting application A and B, except for on the obstacle detection task. A few parameters clearly set lighting application C apart: Spectral Power Distribution (S/P) (A: 1.25; B: 1.22; C: 1.48), CCT (A: 2890; B: 2912; C: 3810) and the light distribution. These parameters will be discussed in relation to the results.

For the perceptual tasks there were significant differences between the lighting applications in the distance needed to identify a facial expression, for reading a street signpost, and the probability of detecting obstacles on the ground. Lighting application C created superior conditions for the perceptual tasks. For obstacle detection, participants were most successful in detecting obstacles under lighting application C, followed by lighting application A, and then lighting application B. For lighting application C, this was in line with both the higher horizontal illuminance level of the targets (E_H A_: 4–8 lx; E_H B_: 2–15; E_H C_: 10–21) stemming from lighting application C’s wider light distribution and with its greater S/P ratio (S/P_A_: 1.25; S/P_B_: 1.22; S/P_C_: 1.48). The peripheral vision used for the task is dependent on the rods, which are most sensitive to light with shorter wavelengths. A light source with a higher S/P-ratio emits a relatively greater amount of light with short wavelengths compared to light sources with a lower S/P-ratio. Light sources with greater S/P-ratio would thus be expected to produce better performance in peripheral tasks, particularly at lower light levels. However, even though lighting applications A and B had similar S/P ratios and similar average horizontal illuminance levels in the obstacle detection area (${\bar{E}}_{H,A}$: 6 lx;, ${\bar{E}}_{H,B}$: 5 lx), the participants were more successful in detecting obstacles under lighting application A. A possible reason for this is the markedly lower illumination values at some of the obstacles for lighting application B, as reflected in uniformity in the obstacle area (U_A_: .59; U_B_: .28). There was also a difference between the two age groups, where the younger group performed significantly better. This is to be expected due to the decline in night vision associated with increased age [57].

Lighting application C enabled the participants to perform their tasks at a significantly greater distance than lighting applications A and B. This could be an effect of the unequal light distribution, which resulted in differences in how much light was reflected off the photograph/sign respectively, with higher luminance levels for lighting application C (Face luminance: *L*_*V A*_: .28; *L*_*V B*_: .36; *L*_*V C*_: .55; Sign luminance: *L*_*V A*_: .13; *L*_*V B*_: .13; *L*_*V C*_: .35). A limitation to this study is that the facial recognition task and sign reading task were conducted in only one location for each task. Had several locations been used, the impact of the different light distributions on the luminance levels of the photographs and the signs would most likely have been clearer.

Once again, the younger group had better results than the older group. For facial expression distance, however, there was also a weak interaction effect indicating that the older group needed to be closest for lighting application B, whereas the shortest distance for the younger group was for lighting application A. This might be due to the group of elderly being more sensitive to glare [58], since lighting application B was more glaring than A, for the distances where most participants recognized facial expressions (approximately 3–10 meters, see Fig 5). For the sign reading test this was not the case. Possibly the location of the task made the participants direct their gaze away from the path, thereby reducing the effect of glare. It is noteworthy, that even though the horizontal illuminance levels for all three lighting applications were well above the recommended levels for pedestrian paths according to the national standards [19], the distances for recognizing facial expressions were much shorter than what is suggested to be the desirable fixation distance (15 meters) [59]. This may be due to the difficulty associated with identifying the facial expressions of black and white photographs, in comparison to real faces.

Lighting application C was rated highest on PSQ and lowest on PCQ for the evaluation aspect. The results on PSQ are not surprising, since lighting application C had the highest mean illuminance (taking the entire laboratory into account) and the greatest S/P ratio. The results for PCQ might stem from the differences in correlated color temperature, since two of the five items constituting the index relate to color temperature (hard-soft and warm-cool). There were also significant differences between the age groups for PCQ, but not for PSQ. The lower ratings of the younger group may represent a greater sensitivity, or stricter standards in the assessment of outdoor lighting. The older group may be more indulgent towards the lighting applications due to their experience of older technologies, such as low-pressure sodium lighting.

The emotional state differed between the different lighting applications on level of arousal, with lighting application C rated highest. This is in line with results from previous research suggesting that lighting applications with a CCT around 5000 K will bring about a greater level of arousal than those with a CCT of around 3000 K, while also being perceived as less pleasurable [47]. In this study, however, there were no significant differences on the valence ratings.

The participants appeared to be able to judge how well they could see under the different lighting applications. Both age groups agreed on lighting application C as the one providing the best seeing conditions. This was in line with their results on the visual tasks, which they performed at a later stage of the experiment. However, the superior seeing conditions did not result in a greater walking speed. It is plausible that the illuminance levels were sufficient for all lighting applications to deliver the visual cues needed for walking at normal pace. The walking speed may also have been influenced by the study being conducted in a laboratory, where participants walked towards the opposite wall and did not have to scan the ground for obstacles. However, another study using a similar setting but with only one lighting application at different levels of dimming, did find significant differences, with walking speed declining with lower illuminances [48].

In this study, lighting application C created superior conditions for the perceptual tasks and was perceived to give the best seeing conditions, indicating that it would be a good choice for pedestrian paths in Malmö. However, at the same time, it was rated as less pleasant on the PCQ subscale of POLQ, and its luminous efficacy is about 68 percent of that of lighting application B. This highlights the fact that there may be no lighting application that is preferable in every given situation. Decision makers therefore need to take the context, as well as the purpose of the lighting, into account and weigh the advantages and disadvantages of lighting applications in order to find optimal solutions.

The study was designed with the intent to assess the human response to outdoor lighting for actual lighting applications used by local municipalities. The aspiration to maximize ecological validity made it difficult to systematically control variables related to the lighting, such as CCT and illuminance level. Naturally, not keeping any variables equal makes it difficult to discriminate which factors were most significant for the difference in perception and evaluation for lighting application C compared to A and B. However, this was never the intention of the study, as the focus was on evaluating three luminaires considered for future use and to collect a set of methods with practical relevance for municipalities in their evaluation of outdoor lighting solutions.

In order to address the three overarching themes of human perception, evaluation and behavior in response to artificial outdoor lighting, this study adopted a broad approach. The methods used were inspired by methods used in previous research and, in some cases, the original method was utilized (POLQ and emotional state). The methods were chosen on the basis of appearing to have high face validity and of being relevant for practice. Both the POLQ scale and the emotional state measure have been shown to be important for the perception of safety, which is a factor that has been frequently discussed in relation to artificial lighting in urban environments.

That outdoor lighting is an important factor, contributing to the walkability of neighborhoods [1–4] and supporting walking for young, adults and elderly [5–11], is well supported. However, pedestrian behavior in the lit environment of real-world settings is less researched. Existing studies have mainly focused on pedestrian flow and walking speed. Walking speed has previously been shown to vary due to different levels of illuminance [48], but in this study the three lighting conditions did not affect walking speed.

However, the use of pedestrian flow and walking speed in real-world settings might be questioned, since the significance of, for example, an increase in walking speed is ambiguous. Do people walk faster due to better seeing conditions or because that the environment is less inviting and perceived as unsafe? Also, pedestrian flow, i.e. the number of pedestrians during a set amount of time, is a crude measure that does not capture how pedestrians move in the lit environment. A supplementary approach could be to simulate pedestrian dynamics based on trajectory data collected from video recordings. This has, for example, been done with regard to pedestrian crossings at signalized intersections [60, 61], but studies modelling the impact of lighting applications on pedestrian behavior are still lacking.

Another approach taken in the study of behavior in the lit environment is to use eye-tracking equipment to capture where pedestrians fix their gaze while walking [24, 62–66]. This might give important insights regarding the relative importance of different visual tasks, as well as indications on how to design light distributions in a better way, but would still need further development to be feasible for large-scale use by municipalities.

There is great potential to cut lighting energy use by upgrading present outdoor lighting installations to more energy-efficient luminaires. Pedestrian experience can be improved and mobility facilitated by upgrading to technologies more adapted to user needs. This study employed methods that differentiated between lighting applications and that could be used to guide the decisions of municipalities before undertaking major upgrades of the outdoor lighting on urban pedestrian paths. We suggest that pilot studies assessing how pedestrians experience the lighting in a real setting can help avoid installing lighting applications with unwanted characteristics, thereby finding a better solution early in the procurement process. We propose that facial expression recognition distance, sign reading distance, the POLQ scale and emotional state measure have potential for use by municipalities in a real-world context. However, validation of these tests and identification of minimum performance thresholds would be needed before considering them to be used as standardized tests.

The obstacle detection task and the motion sensors might be difficult to use outdoors and would need further development, and other methods would be needed to assess pedestrian behavior. One option might be video technology currently used for surveillance purposes. The use of video technology, possibly in combination with eye-tracking, could cast light on where pedestrians direct their gaze and which strategies are employed with regard to glare. Further research is needed to evaluate the methods in the field and to discern the implications of perception, evaluation and behavior for the perceived accessibility and safety, as well as for the choice of walking to different destinations along urban pedestrian paths.

## References

[1] Ewing R, Cervero R. Travel and the built environment. J Am Plann Assoc. 2010;76(3):265–94.

[2] Park S, Choi K, Lee JS. To walk or not to walk: Testing the effect of path walkability on transit users' access mode choices to the station. Int J Sustain Transp. 2015;9(8):529–41.

[3] Kim S, Park S, Lee JS. Meso- or micro-scale? Environmental factors influencing pedestrian satisfaction. Transport Res D-Tr E. 2014;30:10–20.

[4] Park S, Deakin E, Lee SL. Perception-based walkability index to test impact of microlevel walkability on sustainable mode choice decisions. Trans Res Record: J Trans Res Board. 2014(2464):126–34.

[5] Jago R, Baranowski T, Zakeri I, Harris M. Observed environmental features and the physical activity of adolescent males. Am J Prev Med. 2005;29(2):98–104. 10.1016/j.amepre.2005.04.002 16005805

[6] Lee C, Moudon AV. Neighbourhood design and physical activity. Build Res Inf. 2008;36(5):395–411.

[7] Eyler AA, Matson-Koffman D, Vest JR, Evenson KR, Sanderson B, Thompson JL, et al Environmental, policy, and cultural factors related to physical activity in a diverse sample of women: The women's cardiovascular health network project—Introduction and methodology. Women Health. 2002;36(2):1–15. 10.1300/J013v36n02_01 12487137

[8] Addy CL, Wilson DK, Kirtland KA, Ainsworth BE, Sharpe P, Kimsey D. Associations of perceived social and physical environmental supports with physical activity and walking behavior. Am J Public Health. 2004;94(3):440–3. 14998810 10.2105/ajph.94.3.440 PMC1448272

[9] Corseuil MW, Schneider IJC, Silva DAS, Costa FF, Silva KS, Borges LJ, et al Perception of environmental obstacles to commuting physical activity in Brazilian elderly. Prev Med. 2011;53(4–5):289–92. 10.1016/j.ypmed.2011.07.016 21820007

[10] Corseuil MW, Hallal PC, Corseuil HX, Schneider IJC, D'Orsi E. Safety from crime and physical activity among older adults: A population-based study in Brazil. J Env Public Health. 2012;2012(Article ID 641010). 10.1155/2012/641010 PMC3265083 22291723

[11] Rosenberg DE, Huang DL, Simonovich SD, Belza B. Outdoor built environment barriers and facilitators to activity among midlife and older adults with mobility disabilities. Gerontologist. 2013;53(2):268–79. 10.1093/geront/gns119 23010096 PMC3605937

[12] International Energy Agency. Light's labour's lost: Policies for energy-efficient lighting. International Energy Agency; 2006.

[13] Boyce PR, Fotios S, Richards M. Road lighting and energy saving. Lighting Res Technol. 2009;41(3):245–60.

[14] Kuhn L, Johansson M, Laike T, Govén T. Residents’ perceptions following retrofitting of residential area outdoor lighting with LEDs. Lighting Res Technol. 2013;45(5):568–84.

[15] Johansson M, Rosén M, Küller R. Individual factors influencing the assessment of the outdoor lighting of an urban footpath. Lighting Res Technol. 2011;43(1):31–43.

[16] Jägerbrand A, Robertson K, Andersson HB, Folkeson L. Planning and decision-making for more energy efficient road and street lighting in Swedish municipalities Linköping, Sweden: The Swedish National Road and Transport Research Institute (VTI); 2013 Report No.: 786.

[17] CIE. Lighting of roads for motor and pedestrian traffic. Commission Internationale de l’Eclairage; 2010. Report No.: 115:2010.

[18] BSI. Code of practice for the design of road lighting Lighting of roads and public amenity areas. British Standards Institution; 2013. Report No.: BS 5489–1:2013.

[19] Swedish Transport Administration. Krav för vägars och gators utformning, Version 2. Stockholm, Sweden: Swedish Transport Administration; 2015. Report No.: 2015:086.

[20] Lighting Metropolis. www.lightingmetropolis.com [170405].

[21] Rahm J, Johansson M. Walking after dark–A systematic literature review of pedestrians’ response to outdoor lighting. Lund: Dept. of Architecture and Built Environment; 2016. Report No.: 26.

[22] Caminada JF, Van Bommel WJM. New lighting considerations for residential areas. International Lighting Review. 1980(3):69–75.

[23] Dong M, Fotios S, Lin Y. The influence of luminance, observation duration and procedure on the recognition of pedestrians' faces. Lighting Res Technol. 2015;47(6):693–704.

[24] Fotios S, Yang B, Cheal C. Effects of outdoor lighting on judgements of emotion and gaze direction. Lighting Res Technol. 2015;47(3):301–15.

[25] Iwata M, Uchida S. Experiment to evaluate visibility with street luminaires with different upward light output ratios and the use of calculated veiling luminance to determine contrast performance. J Light Vis Env. 2011;35(1):42–54.

[26] Kohko S, Kawakami K, Nakamura Y. A study on affects of veiling luminance on pedestrian visibility. J Light Vis Env. 2008;32(3):315–21.

[27] Fotios S, Castleton H, Cheal C, Yang B. Investigating the chromatic contribution to recognition of facial expression. Lighting Res Technol. 2017;49(2):243–58.

[28] Yang B, Fotios S. Lighting and recognition of emotion conveyed by facial expressions. Lighting Res Technol. 2015;47(8):964–75.

[29] Knight C. Field surveys of the effect of lamp spectrum on the perception of safety and comfort at night. Lighting Res Technol. 2010;42(3):313–29.

[30] Lin Y, Fotios S. Investigating methods for measuring face recognition under lamps of different spectral power distribution. Lighting Res Technol. 2015;47(2):221–35.

[31] Rea MS, Bullough J, Akashi Y. Several views of metal halide and high-pressure sodium lighting for outdoor applications. Lighting Res Technol. 2009;41(4):297–320.

[32] Yao Q, Sun Y, Lin Y. Research on facial recognition and color identification under CMH and HPS lamps for road lighting. Leukos. 2009;6(2):169–78.

[33] Fotios S, Uttley J, Fox S. Exploring the nature of visual fixations on other pedestrians. Lighting Res Technol. 2016(OnlineFirst).

[34] Raynham P, Fotios S. Is facial recognition what matters. Lighting Res Technol. 2011;43:129–30.

[35] Fotios S, Cheal C. Obstacle detection: A pilot study investigating the effects of lamp type, illuminance and age. Lighting Res Technol. 2009;41(4):321–42.

[36] Fotios S, Cheal C. Using obstacle detection to identify appropriate illuminances for lighting in residential roads. Lighting Res Technol. 2013;45(3):362–76.

[37] Uttley J, Fotios S, Cheal C. Effect of illuminance and spectrum on peripheral obstacle detection by pedestrians. Lighting Res Technol. 2017;49(2):211–27.

[38] Fotios S, Uttley J. Illuminance required to detect a pavement obstacle of critical size. Lighting Res Technol. 2018;50(3):390–404.

[39] Johansson M, Pedersen E, Maleetipwan-Mattsson P, Kuhn L, Laike T. Perceived outdoor lighting quality (POLQ): A lighting assessment tool. J Environ Psychol. 2014;39:14–21.

[40] Shikakura T, Kikuchi S, Tanaka T, Furuta Y. Psychological evaluation of outdoor pedestrian lighting based on rendered images by computer graphics. J Illum Eng Inst Japan. 1992;74(10):648–53.

[41] Boyce PR, Eklund NH, Hamilton BJ, Bruno LD. Perceptions of safety at night in different lighting conditions. Lighting Res Technol. 2000;32(2):79–91.

[42] Viliūnas V, Vaitkevičius H, Stanikūnas R, Vitta P, Bliumas R, Auškalnytė A, et al Subjective evaluation of luminance distribution for intelligent outdoor lighting. Lighting Res Technol. 2014;46(4):421–33.

[43] Hanyu K. Visual properties and affective appraisals in residential areas after dark. J Environ Psychol. 1997;17(4):301–15.

[44] Küller R, Wetterberg L. Melatonin, cortisol, EEG, ECG and subjective comfort in healthy humans: Impact of two fluorescent lamp types at two light intensities. Lighting Res Technol. 1993;25(2):71.

[45] Küller R, Wetterberg L. The subterranean work environment: Impact on well-being and health. Env Int. 1996;22(1):33–52.

[46] Quartier K, Vanrie J, Van Cleempoel K. As real as it gets: What role does lighting have on consumer's perception of atmosphere, emotions and behaviour? J Environ Psychol. 2014;39:32–9.

[47] Park N, Farr CA. The effects of lighting on consumers' emotions and behavioral intentions in a retail environment: A cross-cultural comparison. J Interior Design. 2007;33(1):17–32.

[48] Pedersen E, Johansson M. Dynamic pedestrian lighting: Effects on walking speed, legibility and environmental perception. Lighting Res Technol. 2018;50(4):522–36.

[49] Choi J-S, Kang D-W, Shin Y-H, Tack G-R. Differences in gait pattern between the elderly and the young during level walking under low illumination. Acta Bioeng Biomech. 2014;16(1):3–9. 24707805

[50] Itoh N. Visual guidance of walking: Effects of illumination level and edge emphasis. Gerontechnology. 2006;5(4):246–52.

[51] Gustafsson B, Hermerén G, Petterson B. Good research practice Stockholm: Swedish Research Council; 2011. Report No.: 3:2011.

[52] Lin Y, Fotios S, Wei M, Liu Y, Guo W, Sun Y. Eye movement and pupil size constriction under discomfort glare. Invest Ophthalmol Vis Sci. 2015;56:1649–56. 10.1167/iovs.14-15963 25634984

[53] Ekman P. Emotions revealed: Recognizing faces and feelings to improve communication and emotional life 2 ed: New York: Henry Holt; 2007.

[54] Izard CE, Libero DZ, Putnam P, Haynes OM. Stability of emotion experiences and their relations to traits of personality. J Pers Soc Psychol. 1993;64(5):847–60. 8505713 10.1037//0022-3514.64.5.847

[55] Russell JA, Weiss A, Mendelsohn GA. Affect grid: A single-item scale of pleasure and arousal. J Pers Soc Psychol. 1989;57(3):493–502.

[56] Johansson M, Sternudd C, Kärrholm M. Perceived urban design qualities and affective experiences of walking. Journal of Urban Design. 2016;21(2):256–75.

[57] Jackson GR, Owsley C, McGwin G Jr., Aging and dark adaptation. Vision Res. 1999;39(23):3975–82. 10748929 10.1016/s0042-6989(99)00092-9

[58] van den Berg TJTP, van Rijn LJ, Kaper-Bongers R, Vonhoff DJ, Völker-Dieben HJ, Grabner G, et al Disability glare in the aging eye. Assessment and impact on driving. Journal of Optometry, Vol 2, Iss 3, Pp 112–118 (2009). 2009(3):112.

[59] Fotios S, Johansson M. Appraising the intention of other people: Ecological validity and procedures for investigating effects of lighting for pedestrians. Lighting Res Technol. 2017.

[60] Zeng W, Nakamura H, Chen P. A modified social force model for pedestrian behavior simulation at signalized crosswalks. Procedia—Social and Behavioral Sciences. 2014;138:521–30.

[61] Zeng W, Chen P, Yu G, Wang Y. Specification and calibration of a microscopic model for pedestrian dynamic simulation at signalized intersections: A hybrid approach. Transportation Research Part C: Emerging Technologies. 2017;80:37–70.

[62] Davoudian N, Raynham P. What do pedestrians look at at night? Lighting Res Technol. 2012;44(4):438–48.

[63] Fotios S, Uttley J, Cheal C, Hara N. Using eye-tracking to identify pedestrians' critical visual tasks, Part 1. Dual task approach. Lighting Res Technol. 2015;47(2):133–48.

[64] Fotios S, Uttley J, Yang B. Using eye-tracking to identify pedestrians’ critical visual tasks. Part 2. Fixation on pedestrians. Lighting Res Technol. 2015;47(2):149–60.

[65] Fotios S, Yang B, Uttley J. Observing other pedestrians: Investigating the typical distance and duration of fixation. Lighting Res Technol. 2015;47(5):548–64.

[66] Luo W, Puolakka M, Zhang Q, Yang C, Halonen L. Pedestrian way lighting: User preferences and eyefixation measurements. J Lighting Eng. 2013;15(1):19–34.

